# High-Intensity Exercise in Hypoxia: Is Increased Reliance on Anaerobic Metabolism Important?

**DOI:** 10.3389/fphys.2016.00637

**Published:** 2016-12-27

**Authors:** Brendan R. Scott, Paul S. R. Goods, Katie M. Slattery

**Affiliations:** ^1^School of Psychology and Exercise Science, Murdoch UniversityPerth, WA, Australia; ^2^Western Australian Institute of SportPerth, WA, Australia; ^3^New South Wales Institute of SportSydney Olympic Park, NSW, Australia

**Keywords:** metabolic stress, muscle, hypertrophy, sprints, training adaptation

## Introduction

Hypoxic training strategies to optimize physiological exercise responses have been extensively investigated, although often with limited performance benefits over the equivalent normoxic training (Roels et al., 2007). Recently, novel methods including intermittent hypoxic resistance training (IHRT) and repeat sprint training in hypoxia (RSH) have begun to receive research attention. Early results indicate that IHRT can augment muscle hypertrophy and strength compared to normoxic training (Nishimura et al., 2010; Manimmanakorn et al., 2013a,b), while RSH improves fatigue resistance, resulting in an increased capacity for repeated maximal efforts (Galvin et al., 2013; Faiss et al., 2013b). Although performing these high-intensity activities in hypoxia appears to provide some benefits for training adaptations, the mechanisms underpinning these responses are not fully understood. The beneficial responses to high-intensity exercise in hypoxia may result from a greater reliance on anaerobic metabolism, suggesting that increased metabolic stress may drive (or at least contribute to) these adaptations (Faiss et al., 2013b; Scott et al., 2015a). Considering the likely importance of metabolic stress on adaptation to IHRT and RSH strategies, the purpose of this paper is to briefly discuss the potential benefits of high-intensity training in hypoxia with reference to the role of anaerobic processes.

## Impacts of hypoxia on metabolic processes

Both resistance exercise and repeated sprints are characterized by multiple, maximal or near-maximal efforts separated by incomplete recovery periods. Performance during such activities is largely reliant on phosphocreatine (PCr) resynthesis rate (Girard et al., 2011). These high-energy phosphates provide a fuel source during brief high-intensity efforts (Girard et al., 2011), and are therefore necessary to mitigate a decline in performance across repeated efforts. Oxygen availability is an important moderator for PCr resynthesis kinetics, with slower PCr recovery under hypoxic (fraction of inspired oxygen [F_I_O_2_] = 10%) compared with normoxic conditions, and accelerated PCr recovery rates when breathing hyperoxic air (F_I_O_2_ = 100%) (Haseler et al., 1999). During IHRT and RSH, participants recover between efforts in hypoxia, likely impairing PCr resynthesis. Therefore, subsequent efforts are performed under progressively more challenging circumstances, with less energy contribution from PCr stores. This also rationalizes why performing a single set of repeat sprint exercise is relatively unaffected by hypoxic conditions (Goods et al., 2014), whereas impaired performance is observed across multiple sets (Balsom et al., 1994; Billaut et al., 2013; Kon et al., 2015; Morrison et al., 2015).

Exacerbated deoxygenation of skeletal muscle tissue in hypoxia has also been shown to increase reliance on glycolytic rather than aerobic energy production (Bowtell et al., 2014). Although it is not clear regarding the actual contributions of different systems to energy production during IHRT or RSH, research has demonstrated that short duration running performance can be maintained in hypoxia due to a shift toward anaerobic metabolism (Weyand et al., 1999). Corroborating these findings, research investigating both IHRT (Kon et al., 2010, 2012) and RSH (Goods et al., 2014; Morrison et al., 2015) has observed increased concentrations of blood lactate (a by-product of glycolysis) when exercising in hypoxia compared with normoxia. These data suggest that a decrease in oxygen availability during IHRT and RSH increases the contribution of anaerobic metabolism to total energy production, and may result in performance impairment, as first demonstrated by Balsom et al. (1994) over 20 years ago.

Nevertheless, it should be acknowledged that some research has not observed hypoxia to increase markers of metabolic stress during resistance exercise (Kurobe et al., 2015; Yan et al., 2016) or repeat sprint training (Billaut et al., 2013; Gatterer et al., 2014; Goods et al., 2015). In IHRT research, these contrasting findings may result from differences in the structure of exercise. If sufficient repetition volume during sets is not performed, the contraction time during which metabolites accumulate is decreased, and if inter-set rest periods are too long there is a greater chance for intramuscular metabolites to be removed into circulation and PCr resynthesis to occur (Scott et al., 2014, 2015a). Likewise, if adequate recovery is given between efforts during RSH training protocols, the replenishment of PCr stores may occur despite the hypoxic conditions (Girard et al., 2011). Considering that metabolic stress is a likely moderator of adaptation to hypoxic training, optimal training responses may require that exercise and work:rest ratios are structured to exaggerate anaerobic energy production and limit recovery between repeated sets.

## Roles of metabolic stress in adaptations to exercise

Increased metabolic stress is proposed to stimulate various physiological processes associated with muscle hypertrophy (Schoenfeld, 2013). Nevertheless, the exact mechanisms by which this occurs are not fully understood. One possible explanation is an increase in motor unit recruitment. It is possible that metabolic acidosis causes premature fatigue in the fibers initially recruited during exercise, resulting in the activation of additional motor units to maintain the same level of force generation (Manini and Clark, 2009; Manimmanakorn et al., 2013b). This is supported by recent research that has demonstrated heightened integrated electromyography response during moderate-load IHRT compared to the same exercise in normoxia (Scott et al., 2016). If more motor units are recruited during training, a larger portion of the muscle will be stimulated to adapt (Scott et al., 2015a). Cell swelling is another potential mediator for muscle hypertrophy, resulting from metabolite accumulation within the cells and a resultant inflow of water to equilibrate the osmotic gradient (Loenneke et al., 2012). Cellular swelling can increase protein synthesis and decrease protein degradation in a range of cell types (Lang et al., 1998), and it is possible that similar responses occur in muscle cells (Loenneke et al., 2012). Finally, increased growth hormone concentrations have been reported following IHRT protocols (Kon et al., 2010, 2012, 2014), which may be caused by increased lactate build-up and/or metabolic acidosis (Loenneke et al., 2010). However, the role of exercise-induced endocrine responses may not have anabolic effects in healthy individuals as once thought (West and Phillips, 2010), and further research is required to clarify this and other potential mechanisms for hypertrophy during IHRT.

The additional stress associated with hypoxia during repeat sprint training has been demonstrated to improve glycolytic activity in muscle (Girard et al., 2011). This adaptation may be linked to an increased expression of glycolytic enzymes, such as phosphofructokinase (Puype et al., 2013), lactate dehydrogenase (Faiss et al., 2013b) and enzymes involved in pH regulation, such as carbonic anhydrase (Faiss et al., 2013b). Increased metabolic stress can also trigger adaptations that improve pH regulation and enhance blood buffering capacity following repeat sprint training (Girard et al., 2011; Faiss et al., 2013b). Likewise, the creation of a metabolically stressful environment signals adaptive processes that enhance oxygen utilization (via improved blood perfusion) (Casey and Joyner, 2012; Montero and Lundby, 2015) and delivery within the skeletal muscle to facilitate PCr resynthesis during recovery periods (Haseler et al., 1999; Faiss et al., 2013b). Finally, improved fast twitch fiber recruitment similar to that postulated for IHRT is also thought to play a role in enhanced exercise performance following RSH (Faiss et al., 2013a). It is therefore not surprising that the addition of hypoxia to place a further metabolic strain during repeat sprint training has been investigated with promising results regarding performance outcomes. While further research is needed to fully understand how metabolic stress may improve sea-level repeat sprint ability following RSH, the greater metabolic load imposed by hypoxia suggests that it does play an important part in the development of fatigue resistance within skeletal muscle during intense exercise.

## Recommendations and considerations

Considering the apparent importance of metabolic stress on adaptations to IHRT and RSH, the actual exercise performed should be structured with this in mind. For IHRT, substantial repetition volume is likely required during sets to provide sufficient time-under-tension during which metabolic stress can accumulate. Researchers have shown that IHRT causes significant decreases in minimal oxygenation levels in working muscles (Kon et al., 2010), which indicates a more hypoxic intramuscular environment. This would theoretically place more emphasis on anaerobic energy production, increasing the concentration of metabolic by-products (Kon et al., 2010, 2012). If brief inter-set rest periods are implemented, it may be possible to attenuate the clearance of these metabolites, meaning that the next set would begin with already elevated metabolite concentrations within the muscles. Furthermore, brief rest periods would also not allow for PCr stores to be resynthesized to the same levels in hypoxia as in normoxia, whereas extended recovery periods may allow for similar PCr recover, irrespective of hypoxia (Scott et al., 2015a). Current evidence suggests that IHRT should be structured using light to moderate loads (20–70% 1-repetition maximum), which allow for substantial repetitions in each set (10–30 repetitions) and short recovery periods (30–60 s) (Scott et al., 2015a). However, the optimal level of hypoxia to use during IHRT has not yet been determined, and this could obviously have a large impact on training adaptations.

For RSH, an important consideration appears to be the level of hypoxia used, with extreme hypoxic conditions (i.e., F_I_O_2_ ≤ 13%) drastically compromising performance capacity (Goods et al., 2014). From a practical point of view, Gatterer et al. (2014) demonstrated that completing shuttle runs in the confined space of a hypoxic chamber is a viable option to gain the benefits of RSH while maintaining movement specificity. Brocherie et al. (2015) recently used this strategy with success, demonstrating that elite hockey players completing over-ground running RSH in an inflatable hypoxic marquee were able to improve and maintain repeated sprint ability for at least 3 weeks after a RSH intervention. This is critical, as previous researchers have identified limited crossover effects of RSH between cycling and running modalities (Goods et al., 2015). Regarding the actual exercise prescription for RSH, it seems that multiple sets of repeated sprints are required to observe increases in metabolic by-products (Morrison et al., 2015) and longer efforts (>6 s) are likely to have a larger impact on anaerobic metabolism (Faiss et al., 2013b; Puype et al., 2013). Additionally, as proposed for IHRT, short incomplete recoveries via exaggerated work:rest ratios (1:2–1:3) may be more successful for increasing glycolytic stress and therefore performance outcomes (Faiss et al., 2013b) than protocols employing work:rest ratios more commonly used for repeated sprint ability tests (1:5+) (Goods et al., 2015). Nevertheless, it must be acknowledged that some studies have reported varied effects of RSH on metabolic adaptation and performance improvements (Galvin et al., 2013; Brocherie et al., 2015; Goods et al., 2015). Further research is therefore needed to provide conclusive recommendations regarding RSH implementation.

It would also be remiss not to highlight that there are some potential limitations associated with IHRT and RSH strategies. It is possible that the additional stress of hypoxia may result in large performance decrements during training, which could mitigate any hypoxia-mediated benefits from such training. Decreases in concentric velocity during resistance exercise sets has been very strongly related to blood lactate concentration (a marker of anaerobic metabolism; *r* = 0.93–0.97) (Sánchez-Medina and González-Badillo, 2011), and it would therefore be expected that hypoxia-mediated increases in metabolic stress might cause a decline in resistance exercise performance. Nevertheless, our group has not observed hypoxia to impact negatively on performance during high-load resistance exercise (Scott et al., 2015b). Furthermore, several studies have reported an inability to match peak speeds, mean power output or mechanical work performed during RSH compared to normoxic repeat sprint training across an entire session (Billaut et al., 2013; Goods et al., 2014; Morrison et al., 2015). It has also been highlighted that central nervous system fatigue may play a role in decreased performance during hypoxic repeat-sprint exercise, indicating anticipatory central regulation of exercise performance (Billaut et al., 2013). This reduction in performance may attenuate the neuromuscular load of the sprint exercise, and should be considered in the planning and periodization of RSH training. The specific work:rest ratios and accumulated volume to achieve metabolic overload will also be dependent on the prior training status of each individual, which could result in different training adaptations between athletes. Similarly, it is possible that physiological responses to repeat sprint exercise may differ between males and females (Billaut and Bishop, 2009), which could result in altered training adaptations during RSH and should be examined further.

## Conclusions

Adding the physiological stress of hypoxia during exercise essentially makes training more reliant on anaerobic pathways. Recent advances in scientific understanding of IHRT and RSH suggest that this metabolic stress may be an important moderator of adaptations to these novel training strategies, albeit via different mechanisms. It is likely that increased metabolic stress during IHRT causes increased motor unit recruitment and cellular swelling, which may drive (at least partially) hypertrophy of skeletal muscle (Schoenfeld, 2013). During RSH, higher intramuscular concentrations of metabolites may improve glycolytic activity, PCr resynthesis rate, oxygen utilization, and fast twitch muscle fiber behavior, effectively promoting peripheral fatigue resistance (Girard et al., 2011; Casey and Joyner, 2012; Faiss et al., 2013b).

## Author contributions

BS, KS, and PG were involved in manuscript conceptualization, writing, and editing.

### Conflict of interest statement

The authors declare that the research was conducted in the absence of any commercial or financial relationships that could be construed as a potential conflict of interest.
